# Comparing Gene Panels for Non-Retinal Indications: A Systematic Review

**DOI:** 10.3390/genes14030738

**Published:** 2023-03-17

**Authors:** Rebecca Procopio, Jose S. Pulido, Kammi B. Gunton, Zeba A. Syed, Daniel Lee, Mark L. Moster, Robert Sergott, Julie A. Neidich, Margaret M. Reynolds

**Affiliations:** 1 Wills Eye Hospital, Philadelphia, PA 19107, USA; 2 Department of Pathology and Immunology, Washington University/Saint Louis Children’s Hospital, St. Louis, MO 63110, USA; jneidich@wustl.edu; 3 Department of Pediatrics, Washington University/Saint Louis Children’s Hospital, St. Louis, MO 63110, USA; 4 Department of Ophthalmology and Visual Sciences, Washington University/Saint Louis Children’s Hospital, St. Louis, MO 63110, USA

**Keywords:** inherited eye disease, panel testing, congenital glaucoma, retinal dystrophy, congenital cataract, hereditary optic neuropathy, anterior segment dysgenesis

## Abstract

Importance: The options for genetic testing continue to grow for ocular conditions, including optic atrophy, anterior segment dysgenesis, cataracts, corneal dystrophy, nystagmus, and glaucoma. Gene panels can vary in content and coverage, as we and others have evaluated in inherited retinal disease (IRD). Objective: To describe gene panel testing options for inherited eye disease phenotypes and their differences. This review is important for making diagnostic decisions. Evidence review: A licensed, certified genetic counselor (RP) used Concert Genetics and the search terms optic atrophy, corneal dystrophy, cataract, glaucoma, anterior segment dysgenesis, microphthalmia/anophthalmia, and nystagmus to identify available testing options performed by CLIA-certified commercial genetic testing laboratories. Other co-authors were surveyed with respect to genetic panels used for the indications of interest. Ophthalmic panels were then compared using Concert Genetics in addition to their own websites. Findings: Panels from each clinical category were included and summarized. This comparison highlighted the differences and similarities between panels so that clinicians can make informed decisions. Conclusions: Access to genetic testing is increasing. The diagnostic yield of genetic testing is increasing. Each panel is different, so phenotyping or characterizing clinical characteristics that may help predict a specific genotype, as well as pre-test hypotheses regarding a genotype, should shape the choice of panels.

## 1. Introduction

Obtaining a genetic diagnosis for ocular disease involves developing a genetic testing strategy that is comprehensive and cost-effective. In recent years, next-generation sequencing and other molecular and cytogenetic diagnostic modalities have become more accessible and affordable [1,2]. Subsequently, the number of commercial laboratories and options for genetic tests has increased. Gene panels can vary in content and coverage, as we and others have evaluated in inherited retinal disease (IRD) [1,2]. Several options for sponsored or no-charge gene panels are available for IRD, making it easier than ever for clinicians to obtain genetic information. However, panels are quite disparate, and result interpretation is nuanced by these differences.

There is a growing number of genetic testing options for other ocular indications, including optic atrophy and other neuro-ophthalmologic conditions, anterior segment anomalies, cataracts, corneal dystrophy, nystagmus, and glaucoma. Sponsored panels are emerging in these spaces as well, and awareness of the similarities and differences in testing options is important for clinical care and practice. Using the methodology from our previous publication on IRD panels, we sought to compare gene panels for other ophthalmic indications [1].

## 2. Methods

A licensed, certified genetic counselor (RP) used Concert Genetics [3] and the search terms optic atrophy, corneal dystrophy, cataract, glaucoma, anterior segment dysgenesis, microphthalmia/anophthalmia, and nystagmus to identify available testing options performed by CLIA-certified commercial genetic testing laboratories. Additionally, a general internet search was conducted by RP using the same terms. Other co-authors were also asked which genetic panels are used for the indications of interest. Testing options that were not routinely used by the genetic counselor or colleagues were excluded. The websites/pages of the identified panels for the performing laboratories were then visited to collect general information on panel content and coverage, which was recorded when available.

Another co-author who is proficient in pediatric ophthalmology and ocular genetics (MR) collected lists of the genes included on each panel for each of the subgroups noted above. Subsequently, Venn diagrams were made for each subgroup to show the similarities and differences between the distinct gene panels. [4] Finally, as a cross-check of the data, the results for each specialty or indication were then re-evaluated by a co-author who was a specialist in that area (cornea—ZS, glaucoma—DL, pediatrics—KG, optic atrophy—MM, BS). The data were anonymized and presented as CP1 for corneal panel 1, CP2, etc.; GP1 for glaucoma gene panel 1, GP2, etc.; CatP1, CatP2, etc., for cataract gene panels; NysP1, NysP2, etc., for nystagmus gene panels; and OAP1, OAP2, etc., for optic atrophy and neuro-ophthalmologic gene panels.

Overall, sequencing differences between the panels were taken into consideration with respect to understanding how they influenced the interpretation and results. These panels were all “panels” rather than whole exome or whole genome sequencing results.

A descriptive analysis was performed. Each co-author listed the most common indications for which they ordered genetic testing and the most common panels they ordered for specific indications. The most common genetic causes for each disease process that merited genetic testing were recorded [5,6,7,8,9,10,11,12,13,14,15,16,17,18,19,20,21,22,23,24,25]. Whether or not these genes were analyzed by the corresponding panels was then documented.

## 3. Results

**Figure 1:**
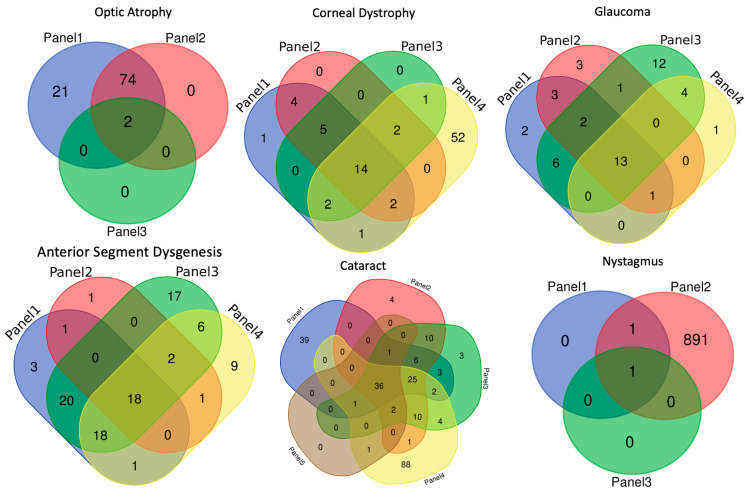
Demonstrates how genes overlap between commercially available panels.

A comparison between the panels from each clinical category is included below and summarized in Table 1 and Figure 1 [4]. This comparison highlights the differences and similarities between panels so that clinicians can make informed decisions.

A. Optic atrophy and Neuro-ophthalmology—Optic atrophy panels may be ordered for individuals with suspected heritable optic atrophy, such as autosomal dominant optic atrophy, or other neuro-ophthalmologic conditions with similar phenotypes, such as Leber hereditary optic neuropathy (LHON). Three panels were included in our analysis. Each panel sequenced 2 to 97 genes from both the mitochondrial and nuclear genomes. Significant overlap with respect to gene inclusion existed between two panels performed by the same lab. One panel focused on optic atrophy-specific phenotypes, while the second panel included a much broader group of neuro-ophthalmologic indications, including LHON, and eye movement disorders, such as nystagmus and progressive external ophthalmoplegia. The remaining panel examined just two genes as well as an unspecified number of variants associated with LHON. However, the two panels, which listed the mitochondrial genes tested by the panel, included the most common 18 allelic variants reported on OMIM. In addition, the most common cause of autosomal dominant optic atrophy is *OPA1*, which was included on all three panels.

B. Corneal dystrophy—Clinicians typically order these panels for patients with suspected corneal ectasia, e.g., keratoconus/corneal dystrophy, e.g., endothelial dystrophies such as Fuchs or stromal dystrophies (such as lattice corneal dystrophy). Four gene panels were used for our comparison. These panels analyze 24 to 75 genes.

First, keratoconus is considered the most common corneal ectasia [5,6]. The gene most commonly associated with keratoconus is *VSX1*. Three of the four panels include this gene. Next, Fuchs endothelial dystrophy has been estimated to affect more than 5% of people over the age of 40 in the USA. Late-onset FECD has been associated with *SLC4A11, ZEB1*, and *AGBL1* [6,7,8,9]. These genes are covered on four, four, and three of the four panels, respectively. Early-onset endothelial corneal dystrophy has been associated with *COL8A2*, which is covered on four of the four panels [10]. Finally, *TGFB1* has been determined to cause lattice corneal dystrophy type 1, Avellino, and Reis–Bucklers. All four panels test for *TGFB1*. However, not all the panels offer full gene sequencing of *TGFB1*. Some test only the most common *TGFB1* pathogenic variants [11].

C. Glaucoma—In our collective experience, these panels are recommended for individuals with congenital glaucoma, juvenile glaucoma, or adult-onset glaucoma with a clear family history. Four panels were used by clinicians and were thus compared. *CYP1B1* and *MYOC* are genetic etiologies for primary congenital glaucoma (PCG) and juvenile open-angle glaucoma (JOAG), respectively, tested by four of the four panels [12,13]. *OPTN* has been associated with primary open-angle glaucoma and is covered by all four panels [6].

D. Anterior segment dysgenesis—This term covers a broad and heterogeneous group of diseases, including developmental disorders that affect the cornea, iris, lens, trabecular meshwork, Schlemm canal, and globe. Patients with these disorders are noted to have iris hypoplasia, small corneal diameter, corectopia, ectopia lentis, and synechiae. Diseases that classically fall under this term include Axenfeld and Rieger anomalies, aniridia, and Peters anomaly. Genetic tests for microphthalmia and anophthalmia, developmental defects of the globe, were also evaluated. Both anterior segment dysgenesis and microphthalmia/anophthalmia can be syndromic or non-syndromic. A summary of common genes associated with these disorders and their representation on our most commonly used panels is in Table 1 [12,14,15,16,17,18].

E. Cataracts—“Free”, or sponsored, testing is now available for patients with bilateral “early-onset” cataracts who are 18 months to 35 years of age. To qualify for testing, cataracts must be bilateral and idiopathic, and patients must live in the US. The “free” testing is an effort to diagnose children with Cerebrotendinous xanthomatosis (CTX), a rare cause of syndromic cataracts due to lipid storage secondary to pathogenic variants in *CYP27A1* gene [19,20]. This syndrome cannot be cured, but its progression is slowed by early treatment with chenodeoxycholic acid (CDCA) replacement therapy, making early diagnosis important. *CYP27A1* is included on four of five panels commonly used by our group.

For those not eligible for “free” testing, the other panels are helpful in analyzing congenital/infantile cataracts diagnosed among younger patients. Congenital/infantile cataracts have both syndromic and nonsyndromic associations. Genetic testing can be beneficial for the early detection of some syndromes to help with screening and management, e.g., galactosemia (*GALT* gene) [21] is included in three of five panels. It can also be beneficial in nonsyndromic cases to help with counseling and other associated eye conditions, e.g., certain genes are associated with glaucoma and cataracts, such as *GJA8* [22] (tested in five of five panels). Other conditions, such as *COL11A1* (included in four of five panels) can be associated with cataracts and Stickler syndrome with an increased risk of retinal detachment [23,24,25].

F. Nystagmus—The differential diagnosis of infantile nystagmus is broad. Genetic testing may be beneficial in the setting of idiopathic infantile nystagmus to narrow the differential and provide prognostic guidance in tandem with a thorough ophthalmologic and systemic examination. In this way, reassurance can be garnered in cases in which clinical examination may be difficult. *FRMD7* is associated with idiopathic infantile nystagmus. The gene panels vary greatly, testing 1 to 893 genes. The more expanded panel tests the causes of congenital decreased visual acuity and central nervous systemic disorders.

## 4. Discussion

While a clinical diagnosis was previously sufficient for patients with many rare diseases, scientific discovery and new advancements in disease diagnosis and treatment have led to an explosion in the need for genetic testing. The availability of testing has been further advanced by the decreased costs of genetic testing and improved insurance coverage. This combination of an increased need for testing and increased access has resulted in an excess number of tests and patients who would benefit from testing with a shortage of skilled providers to order and interpret the results. For ophthalmologists, these needs can be overwhelming [1,2].

First, we must mention the well-described “false discovery” rate. If a panel test with greater than 300 non-mitochondrial genes is ordered, Stone et al. calculated that each patient will have 1.28 plausible disease-causing pathogenic variants [2]. Therefore, careful test selection based on clinical presentation and characterization, i.e., phenotyping, is imperative. The clinical phenotype should generate a hypothesis of the involved genes, and clinicians should review a panel prior to ordering it to ensure that the panel includes these genes. Once results are obtained, clinicians should consider whether the reported results correlate clinically with the patient’s phenotype. Additional post-test work-up and phenotyping may be important considering the results of a genetic test to ensure that a genetic diagnosis is made. Finally, the involvement of skilled ocular and clinical geneticists/genetic counselors, when available, is invaluable.

Next, while it is typically easy to determine genes tested by each panel, the limitations related to the detection of complex genetic aberrations, including rearrangements as well as copy number variants, can be difficult to elucidate. For instance, among cataract panels, one panel notes that deletions of >20 base pairs, insertions or rearrangements of >10 base pairs, and copy number variants of <500 base pairs are not reliable, while another says that deletions and insertions or rearrangements of > 0 base pairs are >99% reliable but offers no data on copy number variants. These factors can be difficult to find on commercial sites, and they influence how one can interpret the results. They are important to consider, as large insertions and deletions as well as single nucleotide variations can result in disease (Table 2).

Additionally, variant interpretation and classification vary between laboratories. The application of the American College of Medical Genetics (ACMG) criteria for variant classification can often only be discerned by prior experience when ordering and receiving reports from a given laboratory. Along the same lines, the pipeline for variant calling also differs, even within the same lab for any given test, which can lead to differences in reporting. Some labs use a phenotype-driven pipeline, while others use a genotype-driven pipeline. Result interpretation should therefore include a thorough review of all available information on a given variant by a genetic counselor or a physician in the context of the patient’s findings.

Finally, this discussion does not include the topic of prenatal diagnosis/preimplantation genetic diagnosis, as our target audience was clinical ophthalmologists, and this complex and important testing usually involves maternal–fetal medicine, reproductive endocrinology and infertility subspecialists, or OB-GYN geneticists. Prenatal diagnosis and preimplantation genetic diagnosis are extremely important topics that merit a review of their own. The role of the ophthalmologist has been previously discussed [26,27]. We recommend that all eye providers discuss this topic with their patients for the purpose of informing them and their families.

## 5. Conclusions

Finally, with all the limitations and the cautionary tales of careful phenotyping, it can be discouraging for ophthalmologists to try to navigate genetic testing. However, the importance of this testing should also not be minimized. Working with a genetic counselor can minimize the burden of test selection, coordination, and follow-up. Access to genetic counselors may be limited, but the importance of their role in providing comprehensive care should be considered.

Testing can be imperative for genetic counseling, diagnosis, screening, and treatment. For example, understanding whether patients have a *WT1* pathogenic variant associated with aniridia can help with Wilms tumor screening and treatment [28]. In addition, there is a growing number of approved treatments and ongoing trials for diseases that require genetic tests. For instance, on clinicaltrials.gov, four treatment trials are actively recruiting patients for Leber’s Hereditary Optic Neuropathy [29].

In summary, access to genetic testing is increasing. The diagnostic yield of genetic testing is increasing. Each panel is different, so phenotyping and pre-test hypotheses should shape the choice of panels.

## Figures and Tables

**Table 1:** Common Phenotype/Genotype associations and panel coverage.

Phenotype	Associated Gene	Included on How Many Panels % (N of Total Panels Compared)
Autosomal dominant optic atrophy	*OPA1*	100% (3)
Leber hereditary optic neuropathy	*MT-ATP6* *MT-CO1* *MT-CO3* *MT-CYB* *MT-ND1* *MT-ND2* *MT-ND4* *MT-ND4L* *MT-ND5* *MT-ND6*	100% (2 that list specific mito genes)
Keratoconus	*VSX1*	75% (4)
Fuchs endothelial dystrophy	*SLC4A11* *ZEB1* *AGBL1* *COL8A2*	100% (4) 100% (4) 75% (4) 100% (4)
Lattice corneal dystrophy—type 1 Avellino Reis–Bucklers	*TGFB1*	100% (4)
Congenital glaucoma	*CYP1B1* *MYOC*	100% (4) 100% (4)
Primary open-angle glaucoma	*OPTN*	100% (4)
Anterior segment dysgenesis 1/Peters anomaly	*PITX3*	50% (4)
Anterior segment dysgenesis 2/congenital primary aphakia	*FOXE3*	100% (4)
Anterior segment dysgenesis 3/iridogoniodysgenesis, Peters anomaly, Axenfeld anomaly, and Rieger anomaly	*FOXC1*	50% (4)
Anterior segment dysgenesis 4/iridogoniodysgenesis or Peters anomaly	*PITX2*	50% (4)
Anterior segment dysgenesis 5/aniridia, Axenfeld and Rieger anomalies, iridogoniodysgenesis, Peters anomaly, and posterior embryotoxon	*PAX 6*	100% (4)
Anterior segment dysgenesis 6/Peters anomaly	*CYP1B1*	50% (4)
Anterior segment dysgenesis 7	*PXDN*	75% (4)
Cerebrotendinous xanthomatosis	*CYP27A1*	80% (5)
Galactosemia	*GALT*	60% (5)
Cataracts/glaucoma	*GJA8*	100% (5)
COL11A1 Stickler syndrome	*COL11A1*	80% (5)
Infantile nystagmus	*FRMD7*	100% (3)

**Table 2:** Panel accuracy for Deletions, Insertions, Rearrangements, Copy Number Variants.

Phenotype	Commercial Panel #	Accuracy of Deletions	Accuracy of Insertions/Rearrangements	Accuracy of Copy Number Variants
Optic atrophy	1	Deletions > 10 base pairs are >99% reliable	Insertions or rearrangements > 10 base pairs are >99% reliable	No data
	2	Deletions > 10 base pairs are >99% reliable	Insertions or rearrangements > 10 base pairs are >99% reliable	No data
	3	No data	No data	No data
Corneal dystrophy	1	No data	No data	No data
	2	No data	No data	No data
	3	Deletions > 10 base pairs are >99% reliable	Insertions or rearrangements > 10 base pairs are >99% reliable	No data
Glaucoma	1	>99% accuracy for deletions < 15 bp in length	>99% accuracy for insertions < 15 bp in length	No data
	2	No data	No data	No data
	3	Deletions > 20 base pairs are not reliable	Insertions or rearrangements > 10 base pairs are not reliable	Copy number variants < 500 base pairs are not reliable
	4	Deletions > 10 base pairs are >99% reliable	Insertions or rearrangements > 10 base pairs are >99% reliable	No data
Anterior segment dysgenesis	1	Deletions > 10 base pairs are >99% reliable	Insertions or rearrangements > 10 base pairs are >99% reliable	No data
	2	Deletions > 20 base pairs are not reliable	Insertions or rearrangements > 10 base pairs are not reliable	Copy number variants < 500 base pairs are not reliable
	3	>99% accuracy for deletions < 15 bp in length	>99% accuracy for insertions < 15 bp in length	No data
	4	No data	No data	No data
Cataracts	1	Deletions > 10 base pairs are >99% reliable	Insertions or rearrangements > 10 base pairs are >99% reliable	No data
	2	Deletions > 20 base pairs are not reliable	Insertions or rearrangements >10 base pairs are not reliable	Copy number variants < 500 base pairs are not reliable
	3	>99% accuracy for deletions < 15 bp in length	>99% accuracy for insertions < 15 bp in length	No data
	4	No data	No data	No data
	5	No data	No data	No data
Nystagmus	1	Deletions > 20 base pairs are not reliable	Insertions or rearrangements >10 base pairs are not reliable	Copy number variants < 500 base pairs are not reliable
	2	Deletions > 10 base pairs are >99% reliable	Insertions or rearrangements > 10 base pairs are >99% reliable	No data
	3	No data	No data	No data

## Data Availability

Available upon request.
